# The role of gangliosides in the organisation of the node of Ranvier examined in glycosyltransferase transgenic mice

**DOI:** 10.1111/joa.13562

**Published:** 2021-10-03

**Authors:** Rhona McGonigal, Hugh J. Willison

**Affiliations:** ^1^ Institute of Infection, Immunity & Inflammation University of Glasgow Glasgow UK

**Keywords:** gangliosides, glycolipid, node of Ranvier, peripheral nerve

## Abstract

Gangliosides are a family of sialic acid containing glycosphingolipids highly enriched in plasma membranes of the vertebrate nervous system. They are functionally diverse in modulating nervous system integrity, notably at the node of Ranvier, and also act as receptors for many ligands including toxins and autoantibodies. They are synthesised in a stepwise manner by groups of glycosyl‐ and sialyltransferases in a developmentally and tissue regulated manner. In this review, we summarise and discuss data derived from transgenic mice with different transferase deficiencies that have been used to determine the role of glycolipids in the organisation of the node of Ranvier. Understanding their role at this specialised functional site is crucial to determining differential pathophysiology following directed genetic or autoimmune injury to peripheral nerve nodal or paranodal domains, and revealing the downstream consequences of axo‐glial disruption.

## Graphical abstract

Gangliosides are a family of sialic acid containing glycosphingolipids highly enriched in the vertebrate nervous system and associated with nervous system integrity and maintenance. In this review we discuss results from the culmination of a series of studies on transgenic mice with varying deficiencies in glycolipids used to dissect the contribution of complex gangliosides and sulfatides to the organisation of the node of Ranvier. This figure shows the likely impact of increasing glycolipid deficiency on paranodal arrangement at nodes of Ranvier based on known data from the peripheral and central nervous systems of transgenic mice.

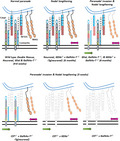

## 1 INTRODUCTION

**FIGURE 1:**
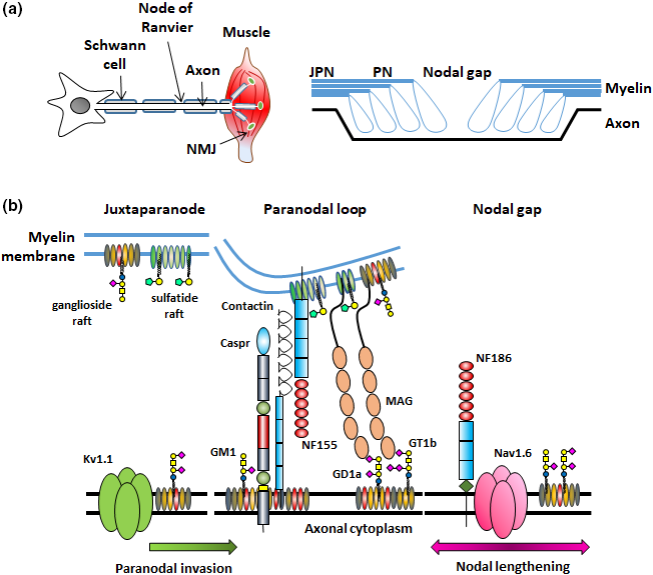
Schematic depicting the peripheral nerve and node of Ranvier. (a) In the peripheral nervous system (PNS) nerves extend from their cell body to their target muscle or organ where they terminate, in this example at neuromuscular junctions (NMJ) on the muscle fibre. Nerves are wrapped in myelin formed by Schwann cells (oligodendrocytes in the CNS). Nodes of Ranvier (NoR) are formed by gaps in the Schwann cells and propagate the action potential. NoR consist of three major domains: the nodal gap is flanked by the paranodes (PN) where the myelin loops attach to the axon. The paranodes in turn are flanked by the juxtaparanodes (JPN). (b) Here, we depict an enlarged representation of a heminode. In each nodal domain there is a special arrangement of axonal and glial proteins that promotes the high‐density clustering of ion channels. Here we depict what is currently understood about glycolipid lipid raft distribution in the nodal membranes and focus on their relationship to key proteins. As such, we recognise that for simplicity we have omitted a number of the proteins within the axons, glia and extracellular matrix that form the NoR. Paranodal axonal proteins Caspr and Contactin are likely highly associated with *a*‐*series* gangliosides and are disturbed in their absence. Interaction with their glial binding partner neurofascin 155 (NF155), forms the paranodal axo‐glial junction. NF155 and myelin associated glycoprotein (MAG) are likely tethered by sulphatide rafts in the paranodal glial membrane. GD1a and GT1b are represented in the axonal paranodal ganglioside rafts as they are the major ligands of MAG, however, in reality the rafts could contain all complex gangliosides. Under protein/lipid deficient or pathological conditions, lengthening of Nav channel domains are indicated by the pink arrow, and invasion of juxtaparanodal Kv channels into the paranode, suggestive of breakdown of the axo‐glial junction at the paranodal/juxtaparanodal border, by the green arrow

Gangliosides are sialic acid containing glycosphingolipids widely expressed in vertebrate plasma membranes and intracellular compartments (Ledeen & Wu, 2011; Sandhoff & Harzer, 2013; Yu et al., 2011). Whilst ganglioside biosynthesis and distribution is widespread throughout the body, the nervous system in particular is very highly enriched in complex gangliosides relative to other organs and tissues. Gangliosides are present in both central and peripheral nervous system (CNS and PNS) grey and white matter, where they have roles in development and homeostasis, modulating cell‐cell recognition, signal transduction, growth and motility. Gangliosides are key components of lipid raft membrane domains where they have been widely studied (Simons & Toomre, 2000; Sonnino et al., 2015). In this review, we will focus our attention on the role of gangliosides in the maintenance of the mammalian node of Ranvier (NoR), a specialised membrane domain formed by myelin‐forming cells: Schwann cells in the PNS and oligodendrocytes in the CNS (Figure 1). The NoR is a critical relay station in the electrophysiological functioning of myelinating nerve fibres, essential for saltatory conduction, promoted by high density clustering of voltage‐gated sodium (Nav) channels on the nodal axolemma. Anchoring of the myelin paranodal loops by adhesion molecules and their associated cytoskeletal linker proteins in both the glial and axonal membranes at the paranode segregate and condense the ion channels (Pinatel & Faivre‐Sarrailh, 2021; Rasband & Peles, 2021). Of note to our review, disruption to the expression of the paranodal axo‐glial membrane protein complex composed of glial Neurofascin 155 (NF155), axonal contactin‐associated protein (Caspr) and Contactin markedly alters the normal conformation of the NoR (Bhat et al., 2001; Boyle et al., 2001; Pillai et al., 2009). A key readout of this includes a lengthening of nodal Nav channel domains and an invasion of juxtaparanodal voltage‐gated potassium (Kv) channel domains into the paranode. Whilst the protein composition at the NoR has been the subject of a wealth of structural and functional studies, the glycolipid/ganglioside component and its effects on nodal integrity has been less extensively studied. The composition and physiology of PNS and CNS nodes have many similarities, but also differences; where relevant we will highlight distinctions between the two sites, but in general refer to the NoR as the generic term for both sites. All the information contained herein refers to the mouse NoR, except where specified otherwise. It has long been known that it cannot be assumed, and has never been systematically proven, that ganglioside distribution biology is identical in all species (Suzuki, 1965), or indeed at all NoR which also differ widely according to site and nerve fibre type.

## 2 GANGLIOSIDE BIOSYNTHESIS

**FIGURE 2:**
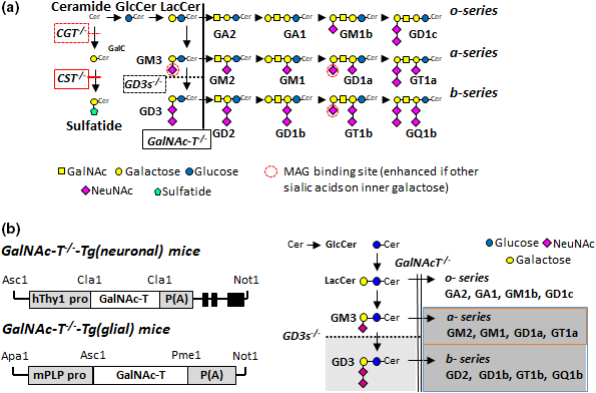
Glycolipid biosynthesis pathways and transgenic modifications. (a) Ganglioside and sulphatide biosynthesis pathways. Gene knock‐outs are indicated and the transgenic lines are defined in boxes. Ceramide is the precursor to galactocerebroside (GalC), sulphatide and gangliosides. The cerebroside sulphotransferase (CST) enzyme synthesises sulphatide from GalC. GalNAc‐transferase (GalNAc‐T) enzyme is required to generate all complex gangliosides while the GD3 synthase (GD3s) enzyme for specific production of *b*‐*series* complex ganglioside. This panel has been modified from Figure 1(a), McGonigal et al. (2019) (https://doi.org/10.1523/JNEUROSCI.2095‐18.2018) with permission under the Creative Commons CC‐BY licence (http://creativecommons.org/licenses/by/4.0/). (b) Constructs (not to scale) were generated to drive GalNAc‐T expression in neurons of *GalNAcT* ^−/−^‐*Tg(neuronal)* mice and in glial cells of *GalNAcT* ^−/−^‐*Tg(glial)* mice. Dark grey shading represents all of the complex gangliosides absent in *GalNAc*‐*T* ^−/−^ mice, while GD3 (light grey) is additionally absent in *GD3s* ^−/−^ *x GalNAc*‐*T* ^−/−^ mice. Promotor activity leads to expression of all complex gangliosides (blue box) in the appropriate membranes in *GalNAcT* ^−/−^‐*Tg(neuronal)* and *GalNAcT* ^−/−^‐*Tg(glial)* mice. In the *GD3s* ^−/−^ *x GalNAc*‐*T* ^−/−^‐*Tg(neuronal)* mice, only *a*‐*series* complex gangliosides are reintroduced on neuronal membranes (orange box). Parts of this panel have been modified from Figure 1(a), Yao et al., (2014) https://doi.org/10.1523/JNEUROSCI.3996‐13.2014 and Figure 2(a), McGonigal et al., (2021) (https://doi.org/10.1111/jnc.15365) with permission under the Creative Commons CC BY‐NC‐SA 3.0 and CC‐BY licences, respectively (http://creativecommons.org/licenses/by/4.0/)

Gangliosides are synthesised in intracellular organelles through stepwise addition of donor sugars to an elongating glycan core, mediated by a family of developmentally and spatially regulated glycolsyltransferases (Yu et al., 2011) (Figure 2a). They are subsequently trafficked to the plasma membrane, then recycled or degraded by lysosomal exoglycosidases. The ability to inactivate glycosyltransferases, through genetic deletion in mice (Figure 2a) or through studying natural human mutations, has led to many of the recent advances in understanding ganglioside biology (Schnaar, 2016). GM3 is the first simple ganglioside synthesised through the addition of α2,3‐linked sialic acid to a lactosylceramide core. GM3 is then modified by the β1,4‐N‐acetylgalactosaminyltransferase 1 enzyme (B4galnt1 or GalNAc‐T) or by the α‐2,8‐sialyltransferase (GD3 synthase, GD3s) enzyme to produce *a*‐*series* or *b*‐*series* gangliosides, respectively. It is GM1 and GD1a from the *a*‐*series*, and GD1b and GT1b from the *b*‐*series* that form the majority of the gangliosides expressed in the normal nervous system. The predominant gangliosides found in neural tissue are thus the complex gangliosides GM1, GD1a, GD1b and GT1b, although many minor gangliosides are also expressed, likely because of high activity of their synthesising enzyme, GalNAc‐T in the nervous system (Dicesare & Dain, 1971). Humans with inherited ganglioside enzymatic deficiency syndromes develop complex neurodevelopment and degenerative disorders (Boukhris et al., 2013; Simpson et al., 2004). Whilst these human disorders display a widespread phenotype, detailed morphological analysis of the NoR has not to date been conducted.

## 3 GANGLIOSIDES AND OTHER GLYCOLIPIDS AT THE NODE OF RANVIER

Apart from their intrinsic functional roles, gangliosides also act as receptors for many microbial pathogens and toxins (Bullens et al., 2002; Ravindran et al., 2013) and peripheral neuropathy‐associated autoantibodies (Kusunoki et al., 2008; Willison & Yuki, 2002). In these roles, the functional effects of ligand binding to gangliosides at NoRs have been determined not to induce pathophysiological perturbations, with the exception of fairly extensive studies on neuropathy‐associated autoantibodies as described below. Knowledge of the anatomical localisation of gangliosides to the NoR first came from studies using ganglioside binding ligands and antibodies, that have now been extensively conducted. Early studies were conducted with the GM1‐binding cholera toxin B subunit and showed localisation on the nodal axolemma and nodal Schwann cell membranes, with scarce detection along the internodal Schwann cell membrane (Ganser et al., 1983). Subsequently a wide range of immunohistological studies with mouse monoclonal antibodies (McGonigal et al., 2010; Paparounas et al., 1999; Susuki et al., 2007a, 2007b) and human antisera (Chiba et al., 1993; De Angelis et al., 2001; Illa et al., 1995; Lugaresi et al., 1997; O'Hanlon et al., 1998; Paparounas et al., 1999) demonstrated immunolocalisation of gangliosides to the nodal gap and paranodal structures, including GM1, GD1a and GQ1b. Much of this research was driven by investigators working on autoimmune neuropathy, in which anti‐ganglioside antibody mediated injury at the node is believed to account in part for the paralytic features of disease (Griffin et al., 1996; Susuki et al., 2012). Since the small size of the nodal region prevents it from being spatially resolved either using mass‐spectrometric imaging or biochemical isolation techniques, it has not been possible to unequivocally identify specific gangliosides in nodal regions, beyond extrapolation from immunolabelling methods. Transgenic mice with disruptions in specific glycosyltransferase genes, and thus deficient in the composition of all downstream gangliosides, have been critical tools in advancing understanding of their roles at NoRs, but since they are generally deleted *‘en bloc’* and subject to precursor build up according to the enzyme position in the biosynthetic pathway, attribution of specific functions to specific gangliosides has also not been possible.

### 3.1 Galactocerebroside and sulphatide

**FIGURE 3:**
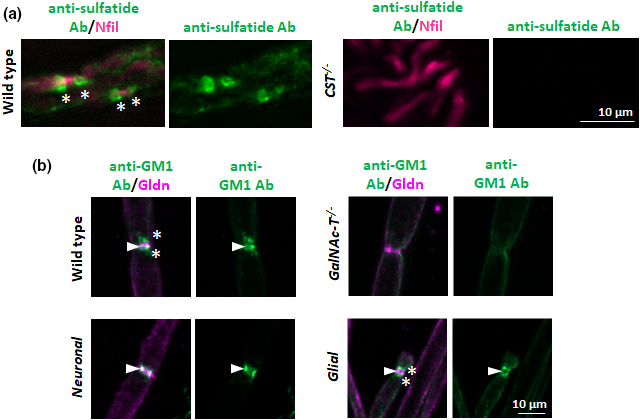
Anti‐sulphatide and anti‐ganglioside antibody binding patterns at the peripheral nerve node of Ranvier. (a) This panel has been modified from Figure 4(a), Meehan et al. (2018) (https://doi.org/10.1016/j.jneuroim.2018.07.004) with permission under the Creative Commons CC‐BY licence (http://creativecommons.org/licenses/by/4.0/). Anti‐sulphatide antibody binding along the myelin and at the paranodal loops (green, *) was observed in the peripheral nerves (magenta) of wild‐type mice, while no binding was detected in CST^−/−^ mice, thus demonstrating the specificity of the antibody to sulphatide. (b) Sciatic nerves were exposed to anti‐GM1 antibody and show selective neural membrane binding among transgenic mice. In wild‐type mice, antibody could be detected at the nodal gap (co‐localised with nodal protein gliomedin (Gldn, arrowhead) and the paranodal loops (*). No antibody staining was detected in ganglioside deficient *GalNAc*‐*T* ^−/−^ mice, demonstrating antibody specificity to GM1. Antibody binding was restricted to the nodal gap in Neuronal nerve (*GalNAcT* ^−/−^‐*Tg(neuronal)* mice), and to the paranodal loops in Glial nerve (*GalNAcT* ^−/−^‐*Tg(glial)* mice), demonstrating the membrane selective expression of complex gangliosides in these transgenic mice. Scale bar = 10 µm

The glycolipids galactocerebroside (Galc) and sulphatide, whilst not gangliosides (as they lack the defining feature, sialic acid), require special mention at the NoR where they have been first and extensively studied in transgenic mice (Coetzee et al., 1996; Dupree et al., 1998; Honke et al., 2002; Hoshi et al., 2007; Ishibashi et al., 2002). Through enzyme knockout technology, the myelin‐localised glycosphingolipids, sulphatide and galactocerebroside were first shown to have a significant role in myelinated nerve maintenance. Their distinct or interdependent roles and interactions are unclear, particularly at the NoR, as GalC deficiency inevitably also leads to sulphatide deficiency which is downstream in the biosynthetic pathway (Figure 2a). Disruption of UDP‐galactose:ceramide galactosyl transferase (CGT) results in mice that are incapable of synthesising either of these lipids; mice thus express a severe dysmyelinating phenotype including NoR disruption, which is more severe in the CNS than PNS (Coetzee et al., 1996; Dupree et al., 1998). Sulphatide, 3‐O‐sulphogalactosylceramide, synthesised from GalC through the addition of a 3‐O‐sulphate group by cerebroside sulphotransferase enzyme (CST), is enriched in the outer leaflet of the myelin and uncompacted glial membranes (Ishizuka, 1997) and small amounts are present in neurons and astrocytes (Eckhardt, 2008). Evidence from *CST* ^−/−^ mice indicates that sulphatides are crucial for the maintenance and stability of the NoR (Honke et al., 2002; Hoshi et al., 2007; Ishibashi et al., 2002). Indeed, in the absence of sulphatide, loss of NF155 immunostaining suggests a role in tethering this key paranodal axo‐glial adhesion molecule to the glial membrane (Hoshi et al., 2007; McGonigal et al., 2019). However, only recently, owing to the development of new anti‐sulphatide antibodies, have immunolabelling studies shown that sulphatide appears highly enriched in paranodal loops, as well as being present along the Schwann cell membrane (Meehan et al., 2018) (Figure 3a).

### 3.2 Complex gangliosides

The advent of glycosyltransferase transgenic mice lacking specific ganglioside species revealed both myelin and nodal disorganisation as a result of different deficiencies, thereby inferentially indicating a role for gangliosides in nodal organisation and maintenance (McGonigal et al., 2021; Susuki et al., 2007a; Yamashita et al., 2005).

The first mouse generated and relevant to the NoR had disruption in the B4galnt1 gene and thus deficiency in GalNAc‐T (Takamiya et al., 1996). These mice are viable and appear grossly normal, indicating that complex ganglioside expression is not necessary for normal development, but later develop an age‐dependent neurodegenerative phenotype characterised by weakness, ataxia, motor deficits, nerve degeneration and demyelination (Chiavegatto et al., 2000; Sheikh et al., 1999; Takamiya et al., 1996). In particular, loss of nodal axo‐glial junction adhesion and integrity, disrupted paranodal loop attachment, invasion of voltage‐gated potassium channels from the juxtaparanode to the paranode coinciding with attenuated paranodal Caspr and NF155 immunostaining at this border was observed (Susuki et al., 2007a). In these mice, overexpression of the precursor simple gangliosides GM3, GD3 and 9‐*O*‐Ac(etyl)‐GD3 may play a compensatory developmental role that limits the severity of the phenotype (Furukawa et al., 2008; Ngamukote et al., 2007). Mice deficient in GD3 synthase and thus lacking *b*‐*series* gangliosides whilst over‐expressing *a*‐*series* gangliosides (*GD3s* ^−/−^) are grossly normal throughout life but repair peripheral nerve poorly (Okada et al., 2002). Nodal abnormalities have not been observed to date in *GD3s* ^−/−^ mice (McGonigal et al., 2010; Susuki et al., 2007a) which could indicate the particular importance of *a*‐*series* gangliosides in maintaining the NoR. Mice whose ganglioside repertoire is restricted to GM3 (*GalNAc*‐*T* and *GD3s* double knock‐out) develop lethal audiogenic seizures (Kawai et al., 2001), age‐dependent progressive motor and cognitive deficits (Tajima et al., 2009), and sensory loss (Inoue et al., 2002). The NoR in GM3 only mice was recently reported to show similar disruptions in nodal immunostaining to those found in *GalNAc*‐*T* ^−/−^ mice (McGonigal et al., 2021), confirming the significance of complex gangliosides to this site as further loss of GD3 added minimal impact. Complete ganglioside ablation is not embryonic lethal; however, from 2 weeks of age, mice undergo progressive and severe neurodegeneration resulting in death at 2 months although the contribution of nodal dysfunction to this has not been assessed (Yamashita et al., 2005). Most ganglioside deficient mice display age‐dependent abnormalities but are not as severely impaired as nodal protein null mice which have a marked reduction in life‐expectancy. Additionally, CNS NoR are often more perturbed than PNS NoR, which could reflect the greater influence that the paranode bestows on CNS stability (Rasband & Peles, 2015). Together, these mouse data are suggestive of a more fundamental necessity for *a*‐*series* gangliosides in age‐related nervous system maintenance, although this is difficult to conclusively prove because a mouse with selective deficiency of *a*‐*series* gangliosides has not been generated.

### 3.3 Gangliosides, sulphatide and myelin‐associated glycoprotein

A specific and interesting issue at the NoR relates to interactions between the glial myelin‐associated glycoprotein (MAG) and gangliosides. The complex gangliosides GD1a and GT1b have been identified as axonal ligands for MAG (Collins et al., 1997; Vinson et al., 2001) (see Figure 2a), localised on the innermost wrap of the myelin sheath, paranodal loops and Schmidt‐Lanterman incisures where glial cytoplasm is present (Bartsch et al., 1989; Trapp et al., 1989). The age‐related degenerative phenotype, functional and morphological deficits displayed by *GalNAc*‐*T* ^−/−^ mice parallel those found in *MAG* ^−/−^ mice, both showing greater severity in the CNS than the PNS. Interestingly, generation of double deficient mice created from interbreeding *GalNAcT* ^−/−^ and *MAG* ^−/−^ genotypes did not further exacerbate the phenotype of the single‐null mice. This suggests a complementary and functional interaction between complex gangliosides and MAG in nerve integrity (Pan et al., 2005). Mice that lack key sialylation enzymes proposed to generate both GD1a and GT1b show a significant reduction in brain MAG immunostaining that coincides with abnormal nervous system development showing the specificity of this interaction (Sturgill et al., 2012). With the knowledge that both MAG and galactolipids modulate axo–glial stability, Marcus et al. (2002) investigated the significance of both molecules to axo–glial integrity by crossing MAG deficient mice with GalC/sulphatide double‐deficient mice, which individually have similar phenotypes. This genetic combination resulted in a lethal phenotype, with survival up to post‐natal day 22 (P22). Again, the NoR appear to develop normally followed by subsequent generalised impairment of the paranodal axo–glial junction.

We advanced these studies to consider the compensatory roles of complex gangliosides and sulphatide in maintaining axo‐glial stability at NoR by combining *GalNAc*‐*T* ^−/−^ and *CST* ^−/−^ strains and examining the nodal phenotype (McGonigal et al., 2019). Depletion of both gangliosides and sulphatide exacerbated the phenotype, suggesting two independent roles. In these animals, a severe neurodevelopmental phenotype occurred with early death around P22. Degenerating axon number increased with diminishing lipid content and conduction became increasingly impaired. MAG expression in the myelin fraction from brain homogenates was significantly lower in the *CST* ^−/−^ x *GalNAc*‐*T* ^−/−^ genotype compared with single deficiency genotypes and Nav channel cluster number, Caspr dimer number, and Nav channels flanked by NF155 dimers decreased with decreasing lipid expression. Once again the nodal phenotype was more severe in CNS compared to PNS nerve. Since MAG acts as a myelin receptor for axonal gangliosides and also may be localised to the myelin membrane by sulphatide‐rich lipid rafts, loss of both the axonal and glial membrane localisation domains for MAG could contribute to this severe phenotype. A progressive reduction in MAG and a significant reduction in NF155 (25% compared to wild type) in *CST* ^−/−^ mouse brains was recently reported (Palavicini et al., 2016), and extraction studies have suggested that sulphatide‐containing lipid rafts could be anchors for MAG and NF155 (Pomicter et al., 2013). Taken together, it seems that glial sulphatide may act as a wide‐ranging anchor for multiple myelin and glial membrane proteins in a complementary and interdependent relationship with axonal gangliosides.

## 4 USE OF TISSUE SPECIFIC TRANSGENIC MICE TO STUDY GANGLIOSIDE FUNCTION AT THE NODE OF RANVIER

Whilst the above studies have indicated that complex gangliosides are required for nervous system maintenance, stability and repair including the NoR, it is unknown whether neuronal or glial ganglioside deficiency has the greater impact on the age‐related phenotype and maintenance of the axon, myelin and axo‐glial junction. Unlike GalC and sulphatide, which are almost exclusively localised to glial membranes, gangliosides are expressed in both glia and neurons (Gong et al., 2002; Ogawa‐Goto & Abe, 1998), and thus function cannot be precisely attributed to tissue expression. An added complexity is that gangliosides can transfer between membranes by shedding and uptake (Heffer‐Lauc et al., 2005; Olshefski & Ladisch, 1996), potentially confounding the concept of discrete membrane localisation. Additionally there is likely to be heterogeneity in ganglioside lipid raft composition within a single membrane; for example distinct separation between GM1‐ and GD3‐containing rafts has been reported (Vyas et al., 2001). To assess the relative significance and necessity of complex ganglioside expression in either neuronal or myelin‐forming cells, we developed “rescue” mice. On a *GalNAc*‐*T* ^−/−^ background, these rescue mice selectively express gangliosides either neuronally [GalNAc‐T being driven by the neurofilament light (NFL) or Thy1 promoters: *GalNAcT* ^−/−^‐*Tg(neuronal)*] or in myelin [GalNAc‐T being driven by the proteolipid protein (PLP) promoter: *GalNAcT* ^−/−^‐*Tg(glial)*](McGonigal et al., 2016; Yao et al., 2014) (Figure 2b). Through this selective reintroduction of glycosyltransferase activity in a site‐specific manner, we observed that neuronal, and not glial, rescue of complex gangliosides was both necessary and sufficient to prevent the age‐dependent neurodegenerative phenotype seen in global *GalNAcT* ^−/−^ deficiency states (Yao et al., 2014). Despite the potential for ganglioside transfer between membranes as mentioned above, we detected no evidence of this transfer between axons and glia, or vice versa in our transgenic rescue mice. Additionally, there was a selective absence of ganglioside expression in primary cultured neurons or glial cells from our *GalNAcT* ^−/−^‐*Tg(glial)* or *GalNAcT* ^−/−^‐*Tg(neuronal)* transgenic lines, respectively, which validated the use of the cell‐specific promotors. These findings clearly demonstrate the prime importance of neuronally expressed GalNAc‐T in maintaining nervous system integrity throughout the lifespan.

With specific reference to the NoR, we considered that, because these glycolipids are differentially expressed in glial and axonal membranes (Figure 3b), they may act in partnership to retain clustered proteins in their respective domains. There is evidence that complex ganglioside deficiency can disturb lipid raft associated anchoring of membrane proteins as has been shown for complement regulators (Ohmi et al., 2011). Axonal Caspr and glial NF155 have been proposed to be in lipid rafts (Schafer et al., 2004), and ganglioside loss disrupts these raft‐associations which coincides with loss of paranodal integrity (Susuki et al., 2007a). We sought to test the importance of specific ganglioside membrane expression by interbreeding strains of single‐null mice and neuronal‐ and glial‐specific rescue mice, thereby allowing us to assess interdependency and cooperativity in the role of axonal and glial glycolipids in paranodal organisation. The expression of *a*‐ and *b*‐*series* gangliosides neuronally reversed the age‐dependent breakdown of the axo‐glial junction in both the PNS and CNS, demonstrated by invasion of Kv channels from the juxtaparanode to the paranode in *GalNAc*‐*T* ^−/−^ mice (Yao et al., 2014). Glial ganglioside expression exacerbated rather than improved the phenotype. It is interesting to note that aged *GalNAc*‐*T* ^−/−^‐*Tg(neuronal)* NoR did not completely return to a wild‐type arrangement; the Nav channel clusters remained lengthened, and the Caspr domains were longer compared to all other genotypes. We further demonstrated that neuronal expression of *a*‐*series* complex gangliosides could promote survival in the *GalNAc*‐*T* x *GD3s* (i.e. GM3‐only) double null mice, but complete restoration to normal would likely require *b*‐*series* expression (McGonigal et al., 2021). In these mice, both CNS and PNS NoR showed only partial recovery to wild‐type composition with the expression of *a*‐*series* complex gangliosides. Shortening of NF155 and Caspr domains indicating loss of axo‐glial integrity was more severe in the CNS and minimally improved. Notably, recovery of nodal protein organisation was dependent on the proteins localisation in either the neuronal or glial membrane. Glial neurofascin 155 was normalised with the expression of neuronal *a*‐*series* gangliosides, likely owing to the contribution of glial sulphatide, but axonal juxtaparanodal Kv channels, Caspr domains and Nav channel clusters remained disrupted, suggesting the need for a contribution from *b*‐*series* gangliosides. This suggests the requirement of specialised lipid raft associated anchoring domains that involve gangliosides. We feel this builds upon the work by Schafer et al. (2004) who proposed the formation of a paranodal lipid raft protein adhesion complex and strengthens the concept that Caspr requires two stabilising mechanisms: protein–protein and protein–lipid interactions that promote stability of the paranodal axo–glial junction (McGonigal et al., 2021).

Rescuing the neuronal, but not the glial, complex ganglioside expression reversed the lethality observed in the *CST* ^−/−^ x *GalNAc*‐*T* ^−/−^ double null mice (McGonigal et al., 2019). Examining the relative importance of *a*‐ and *b*‐*series* gangliosides, we observed that only modest improvement occurred with global *a*‐*series* ganglioside expression on a sulphatide‐null background. Similarly, normal CNS NoR composition in the absence of sulphatide and complex gangliosides could be improved by *a*‐ and *b*‐*series* gangliosides, and not with global *a*‐*series* ganglioside expression. Collectively, these data indicate the importance of neuronal *b*‐*series* gangliosides (e.g., GD1b and GT1b) in maintaining survival in the co‐presence of sulphatide deficiency and indicate interdependency between the functions of these two groups of lipids.

**FIGURE 4:**
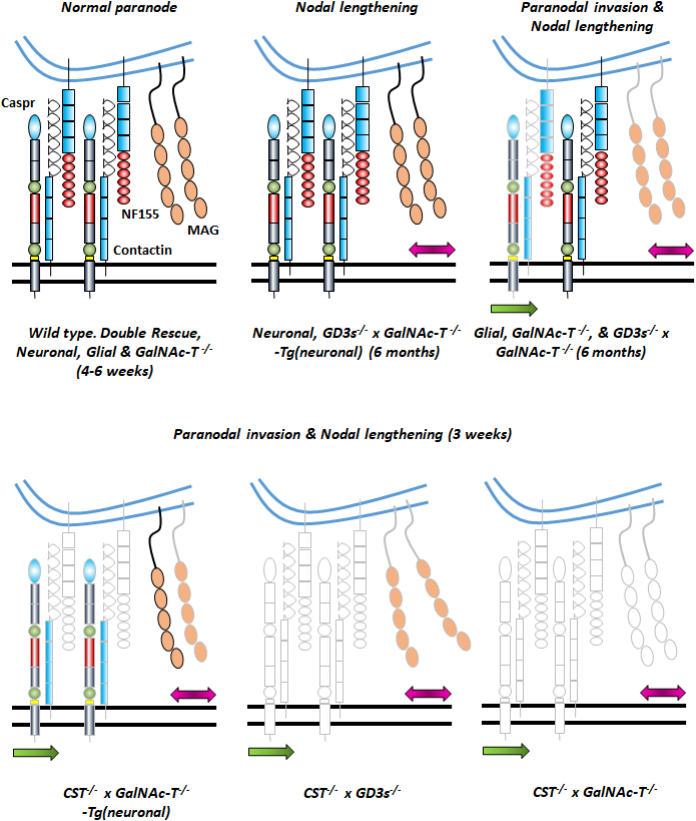
Schematics depicting the likely impact of increasing glycolipid deficiency on paranodal arrangement at nodes of Ranvier based on known data from the PNS and CNS. Particular focus is placed on the paranodal axo‐glial proteins Caspr, NF155 and MAG about which most is known. Lengthening of Nav channel domains are indicated by the pink arrow and invasion of juxtaparanodal Kv channels into the paranode suggestive of breakdown of the axo‐glial junction at the paranodal/juxtaparanodal border, by the green arrow. The transgenic lines represented by each stage of nodal organisation are defined below the relevant schematic, and age is also defined. In ganglioside deficient states, there is nodal lengthening, followed by Caspr/NF155 disturbance and Kv1.1 invasion into the lateral paranode. Normally GD1a and GT1b in rafts will tether MAG but in their absence, MAG does not make the axo‐glial connection. If NF155 is present, this protein can partner with Caspr/Contactin to make an axo‐glial junction. However, when sulphatide is absent, NF155 is also lost from the paranode. In the absence of NF155, Caspr presence is also diminished, and this is exacerbated with the additional loss of complex ganglioside rafts. Under both ganglioside and sulphatide raft deficiency, we propose that MAG and NF155 are both absent from the paranodal axo‐glial domain. This figure uses schematics modified from Figure 8, McGonigal et al., (2019) (https://doi.org/10.1523/JNEUROSCI.2095‐18.2018) with permission under the Creative Commons CC‐BY licence (http://creativecommons.org/licenses/by/4.0/)

These results suggest that the composition of the NoR is largely governed by neuronal *a*‐*series* gangliosides, but that they are not completely sufficient for a normal phenotype. We thus propose that sulphatide and *b*‐*series* ganglioside lipid domains on opposing membranes majorly contribute to a coordinated axo– glial adhesion and paranodal organisation, a combined loss of which leads to severe impairment of nerve integrity with a fatal outcome at an early age. Based on all of the studies discussed, we have created a series of schematics that represent the effect on nodal integrity of progressive glycolipid loss from neural membranes (Figure 4).

## 5 USE OF TISSUE SPECIFIC TRANSGENIC MICE TO STUDY AUTOIMMUNE INJURY TO THE NODE OF RANVIER

As previously published data described in section 4 shows, generation of the rescue mice provides the previously unfeasible opportunity to characterise the contribution of membrane‐specific ganglioside expression on nerve integrity and biological function. Additionally, they provide the ideal system for selectively targeting neural membranes at the NoR and determining the downstream consequences of site‐specific autoimmune injury. Indeed, understanding the NoR pathophysiology of the human disease Guillain‐Barré syndrome (GBS) has been the motivation for generating these mice and the subject of future publications. This anticipates a significant step forward as there exists a few enigmas within the field of inflammatory neuropathies associated with anti‐ganglioside antibodies. Autoantibodies against gangliosides, expressed on both axonal and glial membranes, can be particularly associated with clinical syndromes dominated by specific cellular injury to only one. An example of this is the motor form of the peripheral neuropathy GBS, acute motor axonal neuropathy (AMAN), where motor axons appear to undergo selective injury in comparison with glial membranes (Feasby et al., 1986; Hafer‐Macko et al., 1996). Traditionally, GBS cases are categorised as axonal or demyelinating variants depending on the presumed site of injury. However, it is becoming clear that this rigid classification system does not satisfactorily define patients and their symptoms. For example, patients with nodal dysfunction or disruption can have a spectrum of symptoms ranging from reversible conduction block to axon degeneration leading to quite different prognoses. There has been extensive discussions on the relative contribution nodal membrane injury can impart on nerve function in disease, and the concept of nodo‐paranodopathy versus distinct axonal or demyelinating variants is gaining acceptance as a way to understand the pathophysiological continuum, especially pertaining to anti‐ganglioside antibody driven disease at the NoR (Uncini et al., 2013).

Given the minor nodal abnormalities observed in aged rescue mice, it was essential to characterise the NoR at the age to be used in experimental studies. Herein, we also include data on a further neuronal and glial double rescue mouse. It has recently been reported that circulating anti‐ganglioside antibodies can be sequestered by global ganglioside membrane expression in wild‐type mice (Cunningham et al., 2016) rendering their use for passive immunisation injury studies limited. We observed this finding in wild‐type mice on a C57BL/6 background and do not know if it can be generalised to other strains. Cunningham et al. (2016) showed that this phenomenon does not occur in single neuronal or glial rescue mice, and as such injury models using these mice are possible (McGonigal et al., 2016). It is our expectation that double rescue mice can substitute wild‐type mice where we see no sequestration of anti‐ganglioside antibody, but gangliosides are expressed on both neural membranes. Below we include the nodal characterisation of this newly generated double rescue line compared to the original transgenic mice.

**FIGURE 5:**
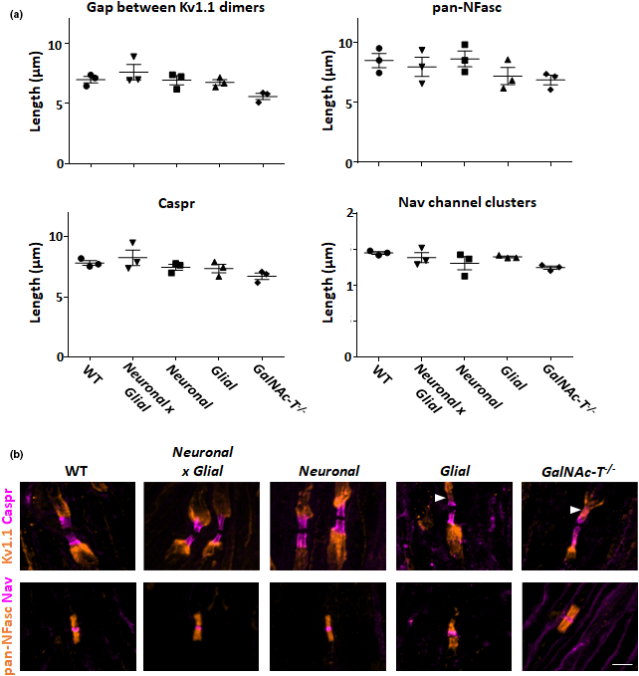
(a) Peripheral nerve nodal organisation is unchanged among mice with selective neural membrane ganglioside expression. Sciatic nerve from 4 to 6 week old wild‐type, Neuronal (*GalNAc*‐*T* ^−/−^‐*Tg(neuronal)*), Glial (*GalNAc*‐*T* ^−/−^‐*Tg(glial)*), Neuronal x Glial, and *GalNAc*‐*T* ^−/−^ mice were analysed (n = 3/ genotype). There were no significant differences among the genotypes in the gap between juxtaparnodal Kv1.1 domains, pan neurofascin (pan‐NFasc, binds both neurofascin 155 and 186) domain length, Caspr domain length or Nav channel cluster length (one‐way ANOVA, *p *> 0.05). (b) Illustrative merged images show immunostaining for: top panel Kv1.1 (orange) and Caspr (magenta); bottom panel pan‐NFasc (orange) and Nav channels (magenta). Caspr immunostaining was located at the paranodes and flanked by juxtaparanodal Kv1.1 in all nerves. Distinct Nav channel clusters were flanked by pan‐Nfasc domains (presumed glial NF155). Paranodal invasion was occasionally observed in Glial and *GalNAc*‐*T* ^−/−^ mice (indicated by arrowhead). Scale bar = 5 µm

At 4–6 weeks of age, there appears to be little detectable impact of membrane selective complex ganglioside expression on nodal organisation (Figure 5). There are no significant differences in the length of Nav channel clusters, Caspr domains, pan‐ neurofascin (pan‐NFasc) domains or the gap between Kv1.1 channel dimers among the genotypes analysed in this study. Direct comparison between wild‐type and *GalNAc*‐*T* ^−/−^ nerve, showed subtle abnormalities in these parameters, also reported previously by Susuki et al. (2007a) at this age. Similar to *GalNAc*‐*T* ^−/−^ mice, we did observe a slight preponderance of glial mice to exhibit mild Kv1.1 invasion into the juxtaparanodal border of the paranode (indicated in Figure 5), and would not recommend their use at older ages as this could indicate the early stages of the disrupted nodal phenotype.

Having established the normal nodal organisation, we assessed the potential for selective nodal targeting by pathogenic antibody. Using a single anti‐GM1 antibody, we show the differential binding patterns at the NoR among wild‐type, *GalNAc*‐*T* ^−/−^, neuronal, and glial ganglioside rescue mice. Figure 3b shows binding at both the nodal gap and paranodal loops in wild‐type mice and an absence of any labelling in *GalNAc*‐*T* ^−/−^ mice. Antibody binding is restricted to the nodal axolemma in neuronal rescue mice and to the paranodal loops in glial rescue mice. These results make these mice ideal for studying the downstream consequences of antibody binding to different neural membranes at the NoR. Knowing that the rescue mice do have an abnormal expression of gangliosides and are prone to show age‐dependent decline in structure, it will be prudent to take this into consideration when interpreting results. However, the current immunohistological data demonstrate that the NoR are grossly normal in all of the lines studied at this age, thereby confirming their suitability for use in peripheral nerve injury models going forward.

## 6 CONCLUSION

Transgenic manipulation of glycosyltransferases has provided a wealth of information on the roles of gangliosides and glycolipids in stabilising specialised membrane domains at the NoR. Whilst knockout studies on many nodal proteins has provided extensive and definitive information on nodal biology, the functional roles of glycolipids are much more complex to investigate and nuanced, owing to redundancy and inability to knockout specific gangliosides. Nevertheless, it is clearly evident that the NoR is rich in glycolipids that function to maintain its integrity and can play a role in pathology. Our rescue mice provide an invaluable tool to explore this field further. It is clear that the NoR is enriched in glycolipids, and whilst this has been the focus of this review, it does not detract from the important and specialised roles gangliosides have in other domains of the nervous system under investigation elsewhere. We anticipate that the legacy of our mice will be their use in exploring and understanding the emerging roles of gangliosides in the nervous system within selective membranes at the NoR and beyond.

## 7 METHODS

### 7.1 Mice

We used 4–6 week old mice, both male and female, from five mouse lines all on a C57BL/6J background: (a) wild‐type; (b) *GalNAc*‐*T* ^−/−^; (c) *GalNAc*‐*T* ^−/−^‐*Tg(neuronal)*; (d) *GalNAc*‐*T* ^−/−^‐*Tg(glial)*; (e) *GalNAc*‐*T* ^−/−^‐*Tg(neuronal*/*glial)*. *GalNAc*‐*T* ^−/−^ mice lack all complex gangliosides globally (Takamiya et al., 1996), while *GalNAc*‐*T* ^−/−^‐*Tg(neuronal)* and *GalNAc*‐*T* ^−/−^‐*Tg(glial)* mice have reconstituted site‐specific expression of complex gangliosides on neuronal (McGonigal et al., 2016) or glial (Yao et al., 2014) membranes, respectively. *GalNAc*‐*T* ^−/−^‐*Tg(neuronal*/*glial)* were generated by interbreeding *GalNAc*‐*T* ^−/−^‐*Tg(neuronal)* and *GalNAc*‐*T* ^−/−^‐*Tg(glial)* mice. Mice were maintained under a 12h light/dark cycle at controlled temperature and humidity with ad libitum access to food and water. Mice were killed by a rising CO_2_ inhalation; all experiments using mice were performed in accordance with a license (POC6B3485) approved and granted by the United Kingdom Home Office and conformed to University of Glasgow institutional guidelines. Experiments complied with relevant guidelines on the care and use of animals outlined in the revised Animals (Scientific Procedures) Act of 1986.

### 7.2 Anti‐ganglioside antibody immunostaining

Sciatic nerves were rapidly dissected and desheathed in oxygenated (95% O_2_ and 5% CO_2_) physiological Ringer's solution containing the following (in mM): NaCl, 129; KCl, 3; NaH_2_PO4, 1.2; CaCl_2_, 2.4; MgSO_4_ 1.3; HEPES, 3; NaHCO_3_, 20 and glucose, 10. Nerves were incubated for 1 h at 4℃ in 100µg/ml anti‐GM1 IgG3 antibody that has been generated as previously described (Boffey et al., 2005; Townson et al., 2007). Nerves were washed and fixed in 4% PFA for 30 min at 4℃, then washed in three 10 min changes of PBS, 0.1 M glycine and PBS. Sciatic nerves were gently teased out into single fibres onto APES coated slides. Slides were incubated overnight at 4℃ in blocking solution (0.3% Triton +3% normal goat serum) with rabbit anti‐gliomedin antibody (Gldn, Abcam #ab24483, RRID:AB_2111616; 1:100). Nerves were washed with PBS followed by application of Alexa Fluor 488‐conjugated goat anti‐mouse IgG3 (Thermo Fisher Scientific Cat# A‐21151, RRID:AB_2535784; 1:500) and Alexa Fluor 555‐conjugated goat anti‐rabbit IgG (Thermo Fisher Scientific Cat# A‐21429, RRID:AB_2535850; 1:500) in PBS with 3% NGS for 2h at R.T. Slides were washed in PBS and mounted in Citifluor.

### 7.3 Nodal Immunostaining

Sciatic nerves (n = 3/genotype) were rapidly dissected into 4% PFA and incubated for 30 min at 4℃. The nerve was washed in three changes of PBS and moved to 30% sucrose for 1 h at 4℃. Nerves were embedded in OCT mount medium and 10 µm longitudinal sections collected onto APES coated slides. Slides were pre‐treated with 100% EtOH at −20℃ for 10 min, thoroughly washed in PBS, before application of a blocking solution (0.3% Triton +10% normal goat serum) for 1 h at 4℃. Slides were then incubated overnight at 4℃ with either one of the following combinations of primary antibodies: rabbit anti‐voltage‐gated potassium channel (Kv1.1, Alomone Laboratories #APC‐009; RRID:AB_2040144; 1:200) plus mouse anti‐Caspr (Antibodies incorporated #75‐001; RRID:AB_ 2083496; 1:300); rabbit anti‐pan neurofascin (anti‐pan‐NFasc; gifted from Professor Brophy, University of Edinburgh, UK; 1:1000) plus mouse anti‐pan voltage‐gated sodium channel (pNav; Sigma‐Aldrich #8809; RRID:AB_477552; 1:100). Following washes in PBS, slides were incubated with secondary antibodies prepared in PBS plus 1% NGS for 2 h at R.T. as follows: Alexa Fluor 555‐conjugated goat anti‐rabbit IgG (Thermo Fisher Scientific Cat# A‐21429, RRID:AB_2535850; 1:500); Alexa Fluor 647‐conjugated goat anti‐mouse IgG1 antibody (Thermo Fisher Scientific Cat# A‐21240, RRID:AB_2535809; 1:500). After PBS washes, slides were mounted in Citifluor.

### 7.4 Imaging and analysis

Images were captured at 40x or 63x magnification using a Zeiss AxioImager Z1 with ApoTome attachment and processed with Zeiss Zen 2 blue edition software. For each staining combination, 5–10 z‐stacks (step value 0.4 µm) were captured per mouse. For peripheral nerve node analysis, images were used to quantify the length of the Nav channel clusters, pan‐NFasc and Caspr domains, and the distance between Kv1.1 dimers from 35 to 90 nodes of Ranvier per mouse. Results were plotted as the average length ±SEM. The tissue was coded upon collection, thereby the experimenter performing analysis was blinded and unaware of the genotype. Statistical differences among genotypes were determined by one‐way ANOVA followed by Tukey's post‐hoc test using GraphPad Prism 6 software (RRID:SCR_002798). Differences were considered significant when *p* < 0.05.

## AUTHOR CONTRIBUTION

Both RM and HJW contributed equally to concept/design, drafting of the manuscript, critical revision and approval of the article. RM acquired the data and performed the data analysis.
